# Data on the effect of Angiotensin II and 6-hydroxydopamine on reactive oxygen species production, antioxidant gene expression and viability of different neuronal cell lines

**DOI:** 10.1016/j.dib.2018.10.069

**Published:** 2018-10-25

**Authors:** Juan A. Parga, Ana I. Rodriguez-Perez, Maria Garcia-Garrote, Jannette Rodriguez-Pallares, Jose L. Labandeira-Garcia

**Affiliations:** a Research Center for Molecular Medicine and Chronic Diseases (CIMUS), University of Santiago de Compostela, 15782 Santiago de Compostela, Spain; b Networking Research Center on Neurodegenerative Diseases (CIBERNED), Madrid, Spain

**Keywords:** 6-OHDA, 6-hydroxydopamine, AngII, angiotensin II, AT1, angiotensin type 1 receptor, AT2, angiotensin type 2 receptor, DCFDA, 2′,7′-dichlorofluorescin-diacetate, H_2_O_2_, hydrogen peroxide, Hmox1, heme oxygenase 1, KLF9, Kruppel like factor 9, MTT, methylthiazolyldiphenyl-tetrazolium, NAC, N-Acetyl-L-Cysteine, Nqo1, NAD(P)H quinone dehydrogenase 1, NRF2, nuclear factor-E2-related factor 2, pLV-empty, pLV-SV40-puro, pLV-KLF9, pLV-SV40-KLF9-puro, ROS, reactive oxygen species, NRF2, KLF9, Dopaminergic, Redox signaling, Oxidative stress, Renin-angiotensin system

## Abstract

This article describes the effect of the oxidative stress inducers Angiotensin II and 6-hydroxydopamine (6-OHDA) on different cell lines. The levels of expression Angiotensin type 1 and type 2 receptors in different dopaminergic cell lines are shown. The data indicate that treatment with Angiotensin II and 6-OHDA increases the production of reactive oxygen species (ROS) and decreases cell viability. NRF2 is a transcription factor induced by ROS. We provide data that NRF2 overexpression increases cell viability in response to oxidative stress inducers compared to control cells, and that these inducers can, both separately and in combination, enhance the expression of NRF2-regulated genes heme oxygenase 1 (Hmox1), NAD(P)H quinone dehydrogenase 1 (Nqo1) and Kruppel like factor 9 (Klf9). Interpretation of these data and additional information is presented in the research article “Angiotensin II induces oxidative stress and upregulates neuroprotective signaling from the NRF2 and KLF9 pathway in dopaminergic cells“ (Parga et al., 2018) [1].

## Specifications table

**Table:** 

Subject area	*Biology*
More specific subject area	*Neuroscience*
Type of data	*Graphs*
How data was acquired	*Plate reader (Tecan Infinite M200), PCR platform (iCicler BIORAD)*
Data format	*Analyzed*
Experimental factors	*Cell lines were treated with AngII and 6-OHDA. Some cells used were modified genetically.*
Experimental features	*Effect of Angiotensin II and 6-OHDA in the ROS production, gene expression and survival of different neuronal cell lines. DCFDA (*2′,7′-dichlorofluorescin-diacetate) *was used as a probe for ROS production. Gene expression was estimated with RT-qPCR. MTT (*methylthiazolyldiphenyl-tetrazolium) *assay was used to analyze cell viability.*
Data source location	*University of Santiago de Compostela, Santiago de Compostela, Spain*
Data accessibility	*Data are available with this article*

## Value of the data

• Data relative to receptor expression in neuronal cell lines could help to understand different responses to Angiotensin II signaling.
• The present data on ROS generation could be used as reference for comparison with those of other cells and stressors to better understand effects of antioxidants or reliability of probes.
• Cell viability can serve as a starting point for using NRF2 to promote cell survival in oxidative stress conditions.
• Gene expression can be used for the design of future experiments targeting the optimal timing for therapies in neurodegenerative diseases and other oxidative stress-associated disorders.

## 1 Data

**Fig. 1:**
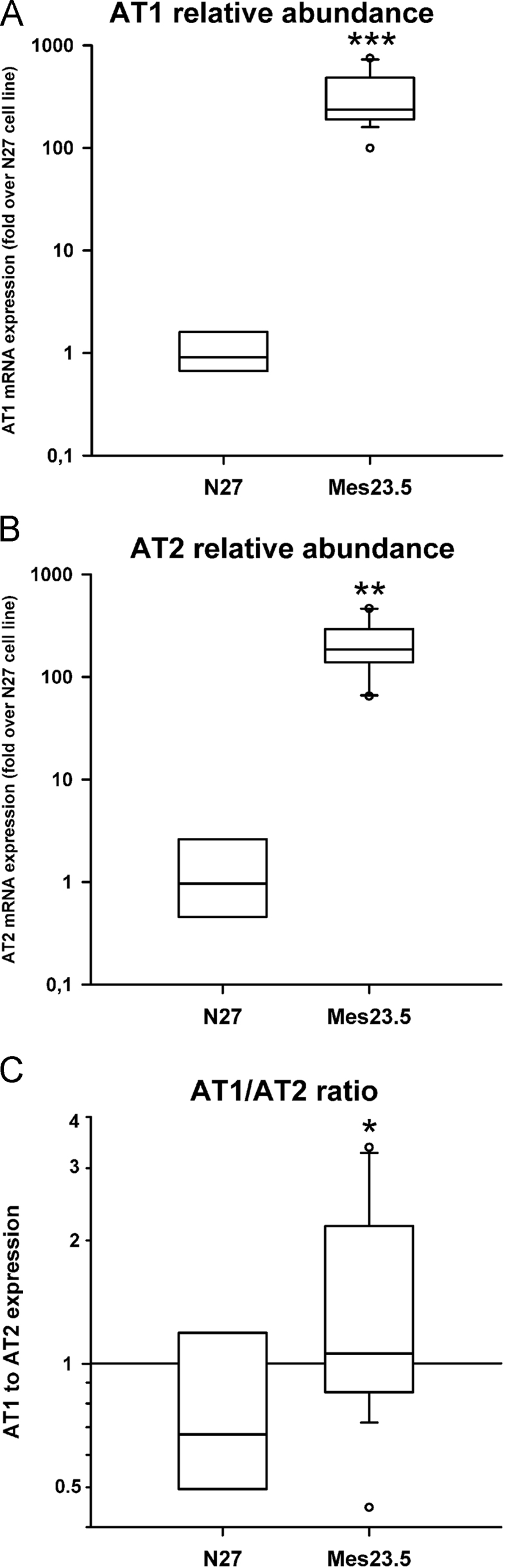
AT1 and AT2 receptor expression is higher in Mes23.5 cells relative to N27 cells. The levels of mRNA of AT1 (**A**) and AT2 (**B**) are higher in Mes23.5 cells compared to N27. Also, there is a relative higher level of AT1 compared to AT2 (**C**). Data are represented as a box plot of the fold increase over N27. *p < 0.05 vs control, **p < 0.01 vs control, ***p < 0.01 vs control. Ang II, Angiotensin II; AT1, Angiotensin type 1 receptor; AT2, Angiotensin type 2 receptor.

**Fig. 2:**
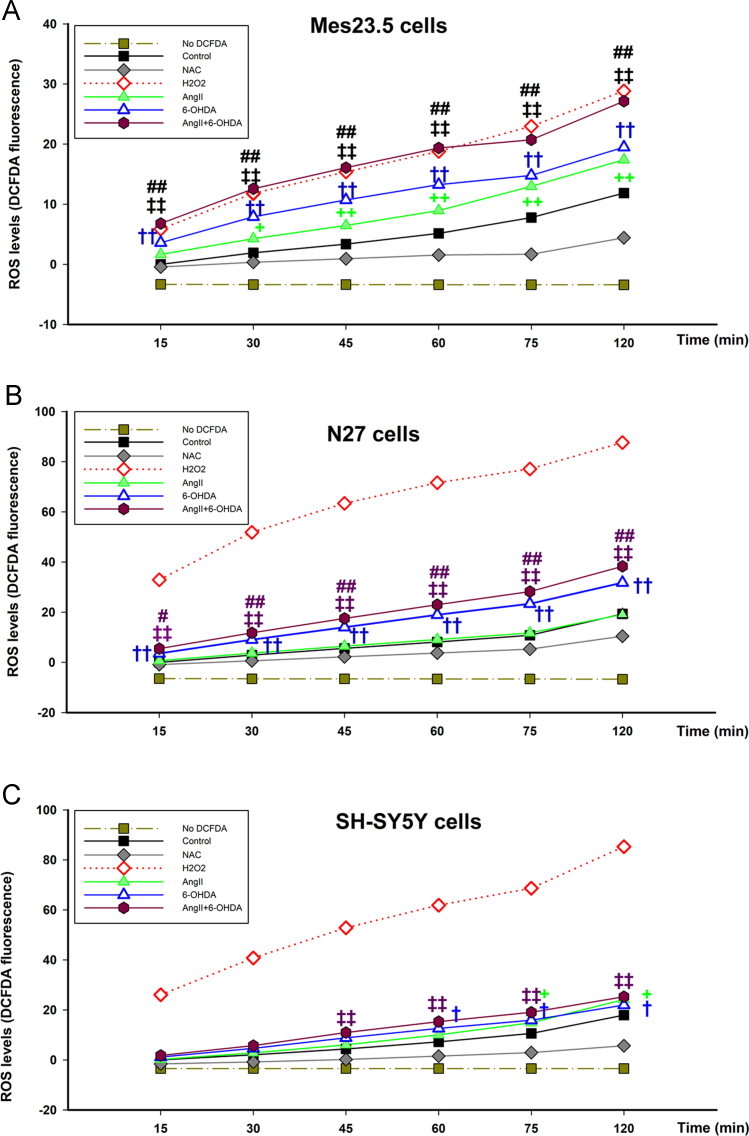
AngII and 6-OHDA increase ROS production in neuronal cell lines over time. Neuronal cells were loaded with probe DCFDA and the effect of different treatments on the ROS production in response to different treatments was measured in a plate reader for 120 min. Graphs show an increase in DCFDA fluorescence over time when dopaminergic Mes23.5 cells are treated with AngII, 6-OHDA or a combination of both (**A**). In dopaminergic N27 cells, treatment with 6OHDA induced an increase in DCFDA fluorescence signal, but no significant effect on ROS production could be measured in the plate reader in response to AngII treatment (**B**) SH-SY5Y cells are sensitive to both AngII and 6-OHDA-induced ROS production, increasing DCFDA fluorescence levels over time (**C**) Data are represented as fold increase in DCFDA fluorescence over control at time 15 min. +*p* < 0.05 AngII vs control, ++*p* < 0.001 AngII vs control; †*p* < 0.05 6-OHDA vs control, ††*p* < 0.001 6-OHDA vs control; ‡*p* < 0.05 AngII+6-OHDA vs control, ‡‡*p* < 0.001 AngII+6-OHDA vs control; #*p* < 0.001 AngII+6-OHDA vs 6-OHDA, ##*p* < 0.001 AngII+6-OHDA vs 6-OHDA. Ang II, Angiotensin II; DCFDA, 2′,7′-dichlorofluorescin-diacetate; NAC, N-Acetyl-L-Cysteine; H_2_O_2_, hydrogen peroxide; ROS, reactive oxygen species.

**Fig. 3:**
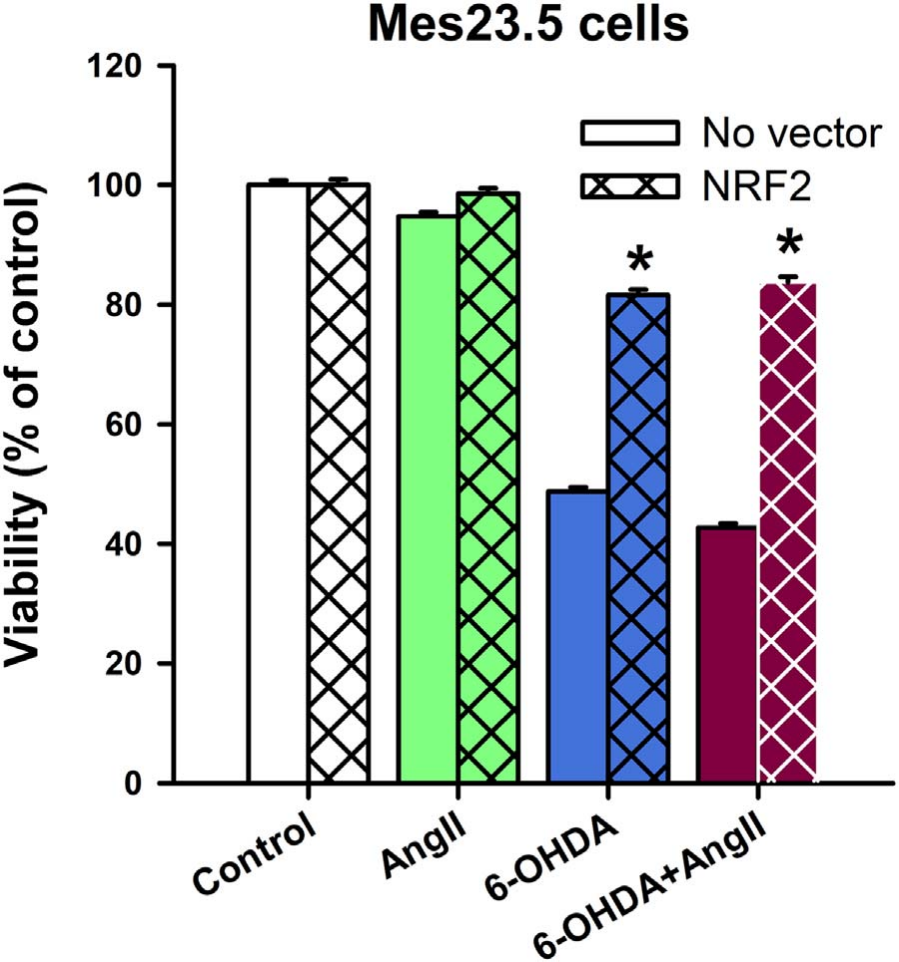
NRF2 expression increases viability in Mes23.5 cells treated with AngII and/or 6-OHDA. Mes23.5 cells were infected by virus carrying a NRF2 and assayed for viability in response to AngII and/or 6-OHDA. NRF2 overexpression increased survival of Mes23.5 in response to oxidative stress inducing agents AngII and 6OHDA. Data are represented as mean absorbance relative to control ± SEM. Values of MTT absorbance of Mes23.5 cells that have not been infected (No vector) from [1] were also included as a reference. *p < 0.001 vs control. Ang II, Angiotensin II.

**Fig. 4:**
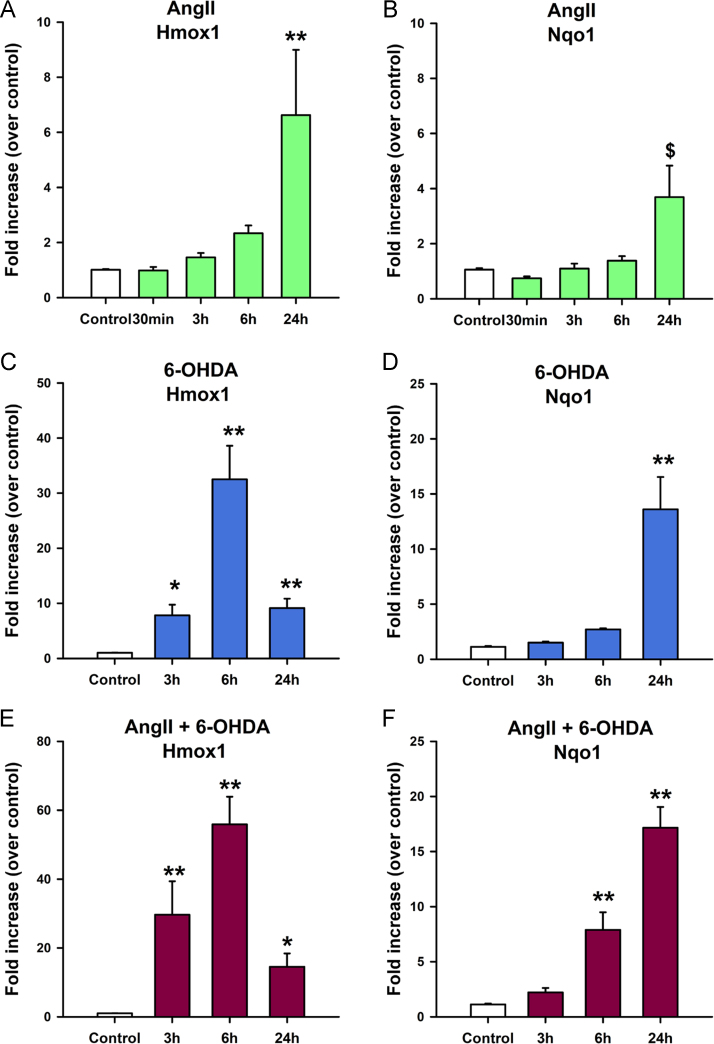
AngII and 6-OHDA increase the expression of Hmox1 and Nqo1 over time. Time course of Hmox1 and Nqo1 mRNA levels in Mes23.5 cells treated with AngII and/or 6-OHDA as measured by quantitative RT-PCR. Treatment with AngII (100 nM) increased Hmox1 (**A**) or Nqo1 (**B**) only after 24 h treatment, when compared to control. 6-OHDA (10 µM), alone or added at the same time as AngII, is a strong inducer of the expression of Hmox1 (**C, E**) that peaked at 6 h post treatment and remained elevated at 24 h. These same treatments induced a marked increase in the expression of Nqo1 at 24 h (**D, F**). Data are represented as fold increase over control ± SEM. *p < 0.05 vs control, **p < 0.001 vs control; $*p* < 0.001 Rank sum test vs control. Ang II, Angiotensin II.

**Fig. 5:**
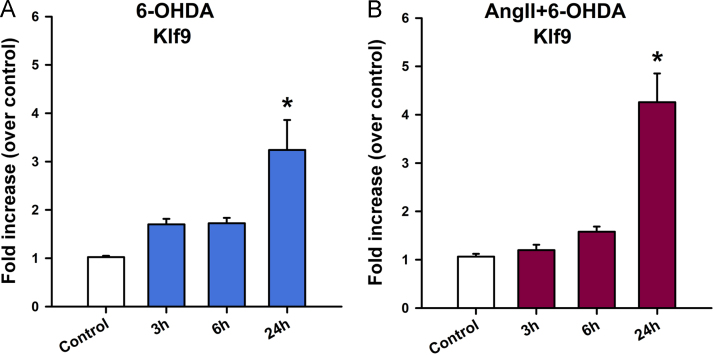
Treatment with 6-OHDA or AngII and 6-OHDA increase the expression of Klf9 after 24 h of treatment. Time course of Klf9 mRNA levels in Mes23.5 cells treated with 6-OHDA and AngII+6-OHDA as measured by quantitative RT-PCR. Treatment with 6-OHDA (**A**) or AngII+6-OHDA (**B**) increased Klf9 only after 24 h treatment, when compared to control. Data are represented as fold increase over control ± SEM. *p < 0.001 vs control. Ang II, Angiotensin II.

**Fig. 6:**
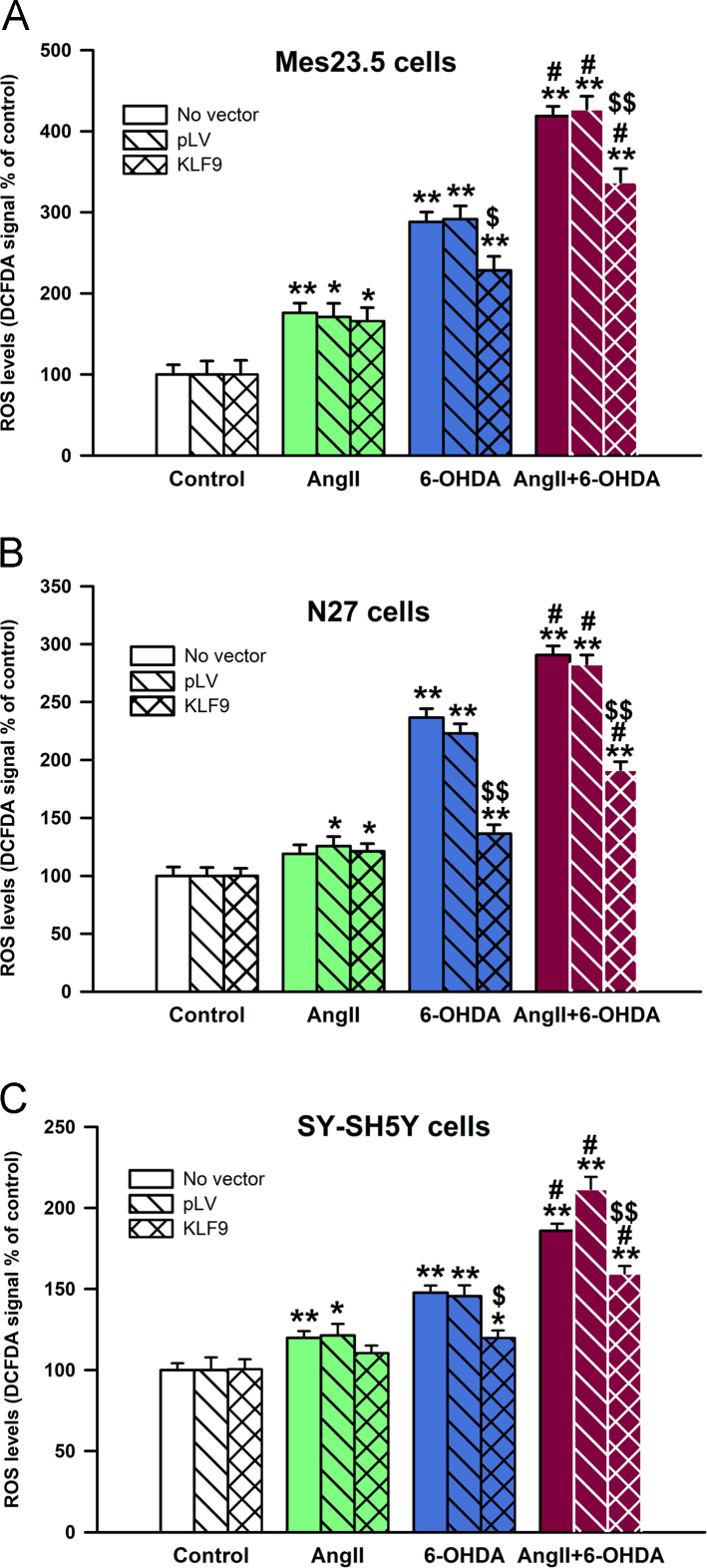
KLF9 expression reduces oxidative stress in different neuronal cell lines. Effect of expression of pLV-empty vector (pLV) and pLV-KLF9 vector (KLF9) on the ROS production was assessed in neuronal cell lines loaded with the probe DCFDA and fluorescence was measured in a plate reader after 60 min. KLF9 expression had no effect on ROS production after 100 nM AngII treatment but greatly reduced ROS levels in response to 6OHDA or a combination of AngII and 6OHDA in Mes23.5 (**A**), N27 (**B**) or SH-SY5Y (**C**) cell lines. Values of DCFDA fluorescence after 60 min of cells from each cell line that have not been infected (No vector) were provided and included for reference purposes. Data are represented as fold increase over control ± SEM. *p < 0.05 vs control, **p < 0.001 vs control; $*p* < 0.05 vs pLV-infected cells, $$*p* < 0.001 vs pLV-infected cells. Ang II, Angiotensin II.

The data presented herein contain gene expression levels of Angiotensin II receptors in dopaminergic cell lines Mes23.5 and N27 (Fig. 1). We also present data on the fluorescence intensity of DCFDA (i.e. ROS generation) over time in these lines and in the human neuroblastoma SH-SY5Y cell line (Figs. 2, 6). We analyzed ROS generation induced by Angiotensin II and/or 6-OHDA, and the corresponding positive and negative controls. Furthermore, we provide data of mRNA expression analysis of genes related to the NRF2 pathway at different time points (Fig. 4, Fig. 5). We also show the effects of NRF2 overexpression on the viability of neuronal cell lines treated with Angiotensin II and/or 6-OHDA (Fig. 3).

## 2 Experimental design, materials and methods

### 2.1 Cell culture

Dopaminergic Mes23.5 cell line, dopaminergic N27 cell line and SH-SY5Y cell line were cultured as previously described [2], [3], [4]. AngII (A9525, Sigma) was used at a concentration of 100 nM 30 min before treatment with the dopaminergic neurotoxin 6-OHDA (0.02% saline ascorbate; H4381 Sigma) when used in combination, except otherwise stated. 6-OHDA was used at a concentration of 10 µM for Mes23.5 or 40 µM for N27 and SH-SY5Y.

HA–NRF2, kindly donated by Dr. Donna D. Zhang (University of Arizona, AZ, USA) was used to infect cell lines. Vectors were transfected into HEK 293 T cells with the corresponding viral packaging plasmids. Viruses were collected from the supernatant and used to infect neuronal cell lines in the presence hexadimethrine bromide (8 µg/ml; H9268, Sigma). Cells were selected with puromycin (10 µg/ml; A11138-03, Thermo Fisher Scientific) starting 72 h after infection. Stable cell lines were established after 2 weeks of selection and were continuously grown in 5 µg/ml of puromycin afterwards.

### 2.2 Cell viability measurement

Cell viability was assessed 24 h after the last treatment: cultures were incubated with MTT (0.5 mg/ml; M2128, Sigma) for 4 h at 37 °C. Culture supernatant was then carefully removed and the formazan crystals resulting from cellular dehydrogenases were dissolved in acidic isopropanol and quantified at 570 nm using a plate reader (Infinite M200, Tecan, Grödig, Austria) after gentle shaking. The absorbances were expressed as percentage of control.

### 2.3 RNA extraction and real-time quantitative RT-PCR

Total ribonucleic acid (RNA) was extracted from cell lines using Trizol (15596-026, Thermo Fisher Scientific). Equal amounts of RNA (2 µg) were reverse-transcribed to complementary DNA with Moloney murine leukemia virus reverse transcriptase (MMLV; 200 U; 28025-013, Thermo Fisher Scientific) following manufacturer׳s instructions. PCR using real-time iCycler PCR platform (Bio-Rad, Madrid, Spain) in combination with iQ SYBR Green Supermix (#1708882, BioRad) were used to assess expression of *Nrf2, Nqo1*, *Hmox1* or *Klf9*, *AT1* or *AT2* using Actin as housekeeping gene. Primer sequences are for rat *Hmox1* F 5′-TCTATCGTGCTCGCATGAAC-3′, R 5′-CCTCTGGCGAAGAAACTCTG-3′; mouse *Hmox1* F 5′-CCGCCTTCCTGCTCAACAT-3′, R 5′-CTGACGAAGTGACGCCATCT-3′; rat *Nqo1* F 5′-GCCCGGATATTGTAGCTGAA-3′, R 5′- GTGGTGATGGAAAGCAAGGT-3′; rodent *Klf9* F 5′-AGTGCATACAGGTGAACGGC-3′, R 5′-AACGGAACTGCTTTTCCCCA-3′; rat *Nrf2* F 5′-CCAGCACATCCAGACAGACA-3′, R 5′-ATATCCAGGGCAAGCGACTC-3′; rodent *Actb* F 5′-TCGTGCGTGACATTAAAGAG-3′, R 5′-TGCCACAGGATTCCATACC-3’; human *HMOX1* F 5′-GAGCCTGAATCGAGCAGAAC-3′, R 5′-AGCCTTCTCTGGACACCTGA-3′; human *NQO1* F 5′-AAAGGACCCTTCCGGAGTAA-3′, R 5′-CCATCCTTCCAGGATTTGAA-3′; human *KLF9* F 5′-GGAAACACGCCTCCGAAAAG-3′, R 5′-GCCGTTCACCTGTATGCACT-3′; human *ACTB* F 5′-GGACTTCGAGCAAGAGATGG-3′, R 5′-AGCACTGTGTTGGCGTACAG-3′. Total RNA (without reverse transcription) was used as a negative control. Relative mRNA expression was calculated by using comparative cycle threshold (2^−ΔΔCt^) method and the relative amount of mRNA was presented in the form of fold change over control.

### 2.4 Measurement of ROS

Cellular ROS measurement was determined using 2′,7′-dichlorofluorescin-diacetate (DCFDA). Cultured cells were washed and loaded with DCFDA (20 µM; D6883, Sigma) for 30 min at 37 °C. Basal fluorescence was measured at this time (time = 0) using a plate reader (Tecan Infinite M200) at excitation 485 nm/emission 520 nm. Fluorescence was again measured 15 min, 30 min, 45 min, 60 min, 75 min and 120 min after addition of different treatments. AngII (100 nM) and 6-OHDA (40 µM) were added at the same time in these experiments. N-Acetyl-L-cysteine (NAC, 600 µM; A7250, Sigma) was used as an antioxidant and control for low ROS generation, and hydrogen peroxide (H_2_O_2_, 100 µM; 1.07209.0250, Millipore) was used as a control for high ROS generation. Fluorescent intensity was expressed as relative units (RU) calculated as the difference from time 0, normalized relative to control at 15 min.

### 2.5 Statistical analysis

All data were obtained from at least three separated experiments, with a minimum sample size of four wells per group and per experiment. Data were expressed as percentage of the control group of the same batch (i.e. they were expressed relative to the value obtained for the control; 100%) and expressed as a mean±standard error of the mean (SEM). Data from *two groups* were analyzed by Mann-Whitney Rank sum test and multiple comparisons were analyzed with one-way ANOVA or two-way ANOVA followed by Holm Sidak test. Differences at *p* < 0.05 were considered as statistically significant. Statistical analyses and graph design were carried out with SigmaPlot 11.0 (Systat Software, GmbH, Erkrath, Germany) except otherwise stated.
