# Supplementary material for: Data on the effect of Angiotensin II and 6-hydroxydopamine on reactive oxygen species production, antioxidant gene expression and viability of different neuronal cell lines
Source: Data Brief. 2018 Oct 25;21:934–42. doi: 10.1016/j.dib.2018.10.069 (PMC6222265; doi:10.1016/j.dib.2018.10.069)
Supplement: Supplementary file 1 — Supplementary material. [file mmc1.pdf]

Data article:

Data on the effect of Angiotensin II and 6-hydroxydopamine on reactive oxygen species production, antioxidant gene expression and viability of different neuronal cell lines

Juan A. Parga , Ana I. Rodriguez-Perez , Maria Garcia-Garrote , Jannette Rodriguez-Pallares, Jose L. Labandeira-Garcia

The corresponding author on the behalf of all authors declare no conflict of interests

October 15, 2018

Signed

Jose L. Labandeira-Garcia MD PhD

LABANDEIRA GARCIA JOSE LUIS - 33223701V

Firmado digitalmente por LABANDEIRA GARCIA JOSE LUIS - 33223701V
Nombre de reconocimiento (DN): c=ES, serialNumber=IDCES-33223701V, givenName=JOSE LUIS, sn=LABANDEIRA GARCIA, cn=LABANDEIRA GARCIA JOSE LUIS - 33223701V
Fecha: 2018.10.15 14:18:15 +02'00'
